# P020: Multiresistant bacteria in positive urocultures in a Dakar university hospital (Senegal)

**DOI:** 10.1186/2047-2994-2-S1-P20

**Published:** 2013-06-20

**Authors:** ML Dia, CT Ndour, A Diop, R Ka, R Diagne, NM Dia, AI Sow, MF Cissé

**Affiliations:** 1 Laboratory of Bacteriology-Virology, CHU of Fann, Dakar, Senegal; 2 Infectious diseases department, CHU of Fann, Dakar, Senegal

## Introduction

Multiresistant bacteria in urines are often associated with nosocomial infections.

## Objectives

The aim of this study was to determine the proportion of multiresistant bacteria in positive urocultures in the Teaching Hospital of Fann.

## Methods

This study was made on data recorded from registers of bacteriological laboratory between 1^st^ January 2008 and 31 December 2011.

## Results

Three hundred and nine multiresistant bacteria (309) among the 709 mutiresistant strains were isolated from urines (43,58 %). The mean age was 39,73 years [range=1 – 83] with a sex ratio of 0.88. Most of the patients were hospitalized (62,5 %). The infectious diseases clinic provided most of the multiresistant bacteria (41,1 %), followed by the neurology department (14,24 %) and paediatrics department (12,23 %). The majority of multiresistant bacteria were constituted by extended spectrum betalactamase enterobacteriaceae (86, 08 %) and Acinetobacter spp (5, 50 %). E. coli were the most frequent bacteria (35, 92 %) followed by Klebsiella pneumoniae (35, 60). Enterobacteriaceae were susceptible to imipenem, amikacin and colistin but were resistant to quinolones and other aminosides. Methicillin-resistant Staphylococcus aureus and methicillin-resistant Staphylococcus saprophyticus were susceptible to vancomycin. Strains of Acinetobacter were susceptible to imipemem and colistin.

## Conclusion

Most of the multiresistant bacteria in the teaching hospital of Fann are isolated from urines. That’s why it is important to insist on prevention by respecting hygiene measures during invasive gestures like pose of urinary catheters.

## Disclosure of interest

None declared

